# Atheists and Agnostics Are More Reflective than Religious Believers: Four Empirical Studies and a Meta-Analysis

**DOI:** 10.1371/journal.pone.0153039

**Published:** 2016-04-07

**Authors:** Gordon Pennycook, Robert M. Ross, Derek J. Koehler, Jonathan A. Fugelsang

**Affiliations:** 1 Department of Psychology, University of Waterloo, Waterloo, Canada; 2 Department of Psychology, Royal Holloway, University of London, London, United Kingdom; 3 ARC Centre of Excellence in Cognition and its Disorders, Macquarie University, Sydney, Australia; University of Amsterdam, NETHERLANDS

## Abstract

Individual differences in the mere willingness to think analytically has been shown to predict religious disbelief. Recently, however, it has been argued that analytic thinkers are not actually less religious; rather, the putative association may be a result of religiosity typically being measured after analytic thinking (an order effect). In light of this possibility, we report four studies in which a negative correlation between religious belief and performance on analytic thinking measures is found when religious belief is measured in a separate session. We also performed a meta-analysis on all previously published studies on the topic along with our four new studies (*N* = 15,078, *k* = 31), focusing specifically on the association between performance on the Cognitive Reflection Test (the most widely used individual difference measure of analytic thinking) and religious belief. This meta-analysis revealed an overall negative correlation (*r*) of -.18, 95% CI [-.21, -.16]. Although this correlation is modest, self-identified atheists (*N* = 133) scored 18.7% higher than religiously affiliated individuals (*N* = 597) on a composite measure of analytic thinking administered across our four new studies (*d* = .72). Our results indicate that the association between analytic thinking and religious disbelief is not caused by a simple order effect. There is good evidence that atheists and agnostics are more reflective than religious believers.

## Introduction

Dual-process theories distinguish between two fundamentally different types of cognitive processes [1]: Type 1 processes that are intuitive and autonomously cued and Type 2 processes that are reflective and require working memory. One of the most important insights that has emerged from the dual-process literature is that the distinction between intuition and reflection is of consequence to more than just researchers interested in thinking and reasoning [2]. For example, the propensity to engage analytic reasoning (as distinct, conceptually, from cognitive ability) predicts paranormal disbelief [3, 4], acceptance of science [5, 6], less traditional moral values and judgments [7, 8], less reliance on Smartphones as an external source of information [9], and a lowered receptivity to bullshit [10]. These results indicate that the interplay between intuitive and analytic processes is an important component of human cognition. The degree to which analytic (Type 2) processes influence reasoning and decision making in lab studies is at least somewhat predictive of specific theoretically grounded real world outcomes [2].

Most importantly for the present investigation, there is now a good deal of evidence that analytic thinking disposition (“analytic cognitive style”) is negatively associated with religious belief [11]. For example, religious believers perform worse than non-believers on cognitive tests that cue incorrect intuitive responses [4, 12, 13]. Consider, for instance, the bat-and-ball problem from the Cognitive Reflection Test (CRT; [14]): “A bat and a ball cost $1.10 in total. The bat costs $1.00 more than the ball. How much does the ball cost?” The majority of participants respond “10 cents” to this question (e.g., 64.9% in [15]). This is the response that typically comes to mind upon an initial read of the problem, but it is incorrect. If the ball is $0.10, then the bat must be $1.10 and in total they would be $1.20. To recognize that the intuitive response is incorrect, the participant must be willing to stop and think analytically about the problem despite having what seems to be a plausible intuitive response. Performance on the CRT is therefore thought to index, at least to some degree, a willingness or propensity to engage Type 2/analytic processing [14, 16, 17, 18]. Naturally, it also requires some degree of cognitive ability (and, in particular, numeracy) to solve the arithmetic involved in each of the problems [19]. As a consequence, a number of studies have also demonstrated that there is a negative association between religious belief and CRT performance (and performance on related tasks) even after controlling for measures of cognitive ability and intelligence ([4, 7, 13, 20], but see [21]). This negative association also remains robust in regression analyses that control for various demographic factors (e.g., age, sex, education; see [11] for a review). Furthermore, religious believers spend less time reasoning when given problems in a lab study [20, 22], as would be expected if they are less willing to engage slow, deliberative reasoning processes. Finally, there is experimental evidence that inducing an analytic or reflective mindset at least temporarily decreases self-reported religious belief [12, 13, 23].

These results were challenged in a recent paper by Finley, Tang, and Schmeichel [24]. Specifically, they hypothesized that the association between analytic thinking and religious belief depends on the order in which analytic thinking and religious belief measures are presented. In a high powered experiment, the authors replicated the negative association between religious belief and CRT performance when the CRT was administered immediately prior to the religious belief measure. However, they did not find an association when the CRT was administered after the measure of religious belief. This is a potentially important finding because many of the previously cited studies measured analytic thinking before religious belief (e.g., [4, 7, 12, 20]).

Importantly, Finley et al. do not question the claim that analytic thinking decreases religious belief, which is supported by experimental evidence [12, 13, 23]. Rather, they argue that “more analytic thinkers are not necessarily less religious” (abstract, p.1). According to Finley et al., the idea that analytic thinking decreases religious belief at the state level does not necessarily contradict the claim that they are not associated at the trait level. Experiments in which analytic thinking decreases religious belief serve only as an existence proof–it is still quite possible that analytic thinking is not a meaningful component of religious cognition in most people’s everyday lives. In other words, the modal analytic thinking disposition may not be sufficiently analytic to be of consequence for religious belief. This line of reasoning seems to contradict the wealth of data that indicates that analytic thinking does have consequences for our everyday lives (as summarized above; see [2] for a review), but religious belief may be an exception.

Finley et al. argue that asking participants to first indicate their religious belief and then answer reasoning problems is the purest way to test whether there is a genuine association between the two variables. In support of this contention, the authors point to previous research wherein moral judgment was only associated with CRT performance if the CRT was given prior to the moral judgment task [25]. It may be that the CRT (and, presumably, related measures) induces an analytic mindset, which then somehow interacts with self-reported religious belief. Finley et al. are, unfortunately, not entirely clear about why they make this prediction. They state that (p. 2): “By having participants complete the CRT prior to reporting religious beliefs, Gervais and Norenzayan likely activated an analytic mindset in participants who answered analytically (i.e., correctly), temporarily suppressing religious beliefs. As a result, the strength of the relationship between individual differences in analytic thinking and religious beliefs may have been inflated by first activating the analytic system, rather than representing a pure (i.e., confound free) relationship between individual differences in analytic thinking and religious beliefs.” Finley et al. qualify their claim by arguing that only those participants who are particularly analytic in the first place would be put in an analytic mindset by the CRT. However, if this is the case, only those participants who have religious beliefs in the first place would have their religious beliefs suppressed. Presumably, then, the differences between religious disbelievers (those scoring near zero on a religious belief measure) and the various levels of religious believers would decrease if the CRT is presented first. Or, perhaps, there is a more complex interaction that requires a necessary level of analytic cognitive style for the CRT to induce an analytic mindset and that only affects some levels of religious belief and not others. These speculations illustrate that the mechanisms proposed by Finley et al. are not at all straightforward. Even if the association between CRT and religious belief is only evident when religious belief is measured first, it is not clear why this would be the case.

Given this uncertainty, the key question is whether one or the other presentation order is more likely to reflect the actual association (or lack thereof) between analytic thinking and religious belief. There is some extant data that indicates that, contrary to Finley et al.’s claims, the apparent lack of correlation when religious belief was measured first in their study is the *exception*, not the rule. First, six published studies (2 of which were published before Finley et al.) had participants indicate their religious belief prior to solving the CRT and found significant associations [26, 27, 28, 29]. In each case, however, the additional tasks were included in-between the religiosity measure and the CRT. This may explain the discrepancy between these studies and Finley et al. (although, of course, they cannot be accounted for given Finley et al.’s hypothesis about scale administration order).

Second, there is evidence for the association from large surveys with intervening tasks separating analytic thinking and religious belief measures (e.g., [7, 10, 13, 30]). Finley et al. disputed this evidence by suggesting that these surveys may have included other measures that could have induced an analytic mindset. We are not convinced. A number of very different intervening tasks have been used, including, for example, a moral values questionnaire [7], a bullshit receptivity scale and ontological confusions measure [10], and demographic questions [30]. Regardless, it is possible that the CRT induces an analytic mindset that persists during these intervening tasks.

Third, some investigations of analytic thinking and religiosity have included categorical variables that should, in theory, be less susceptible to contextual or experimental modification. For example, Pennycook et al. [4] found an association between CRT performance and the *type* of God belief (or disbelief). Namely, those who believed in a conventional “personal” God scored the lowest and those who lacked any belief in God (i.e., atheists) scored the highest on the CRT. It seems unlikely that an analytic mindset would be sufficient to make a personal God theist into an atheist in the context of a single study session.

Finally, Pennycook et al. [22] did not measure religious belief in the same study session as analytic thinking and nonetheless found a negative association between the variables. This is inarguably the purest way to investigate a possible association. For this, Pennycook et al.’s participants completed a religious belief scale in a separate “mass testing” survey that opened for participation at the beginning of the semester. The mass testing survey included a number of scales that were submitted by numerous research groups in the same department (e.g., boredom proneness, a Big Five personality scale), but no analytic thinking measures. Importantly, the order of the scales was randomized for each participant, with the exception of a boredom proneness questionnaire that always came first and an aggression questionnaire that always came last. In this case, religious belief was measured prior to analytic thinking and, more importantly, in the context of an entirely different study.

Pennycook et al. [22] also reported an association between response time on a base-rate neglect task and self-reported religious affiliation. This question was completed in a “pre-screen” questionnaire that was separate from both the analytic thinking tasks and the religious belief scale (Note that although the pre-screen questionnaire is typically used for data screening, we did not restrict participation in the discussed study [22] or in any of the new studies reported here). For this, participants simply selected which of a series of religious affiliations or disaffiliations they most strongly identified with (e.g., Christian, Muslim, Agnostic, Atheist, None). The pre-screen questionnaire only included demographic questions of this sort and the religious affiliation question came after questions about ethnic identification. Regardless, as mentioned above, it seems unlikely that an analytic mindset (if it were somehow present) would cause someone who identifies as a Christian to suddenly identify as an atheist (for example).

These results represent a strong challenge to Finley et al.’s claim that there is no genuine association between analytic thinking and religious disbelief. It is unlikely that participants were in a particularly analytic mindset when indicating their level of religiosity in the mass testing and pre-screen surveys, yet the association was present in each of these studies. Nonetheless, it may still be the case that “the link between analytic thought and religious belief is more tenuous than previously reported” (p. 1). As such, we report four additional studies in which analytic thinking and religious belief were measured in separate sessions. In these studies, religious belief was measured in a mass-testing survey administered to university students at the beginning of four different academic semesters. Participants also indicated their religious affiliation (or lack thereof) in a separate pre-screen questionnaire. Participants who completed these questionnaires were then permitted to sign up for an online study with a battery of cognitive tasks. However, when this permission was granted there was no connection made between the online study and the mass testing or pre-screen surveys (i.e., the online study was referred to as a “Thinking Styles and Reasoning” study and neither the mass testing nor pre-screen sessions were mentioned). Finding a negative correlation between performance on analytic thinking and religious belief and affiliation across each of the four studies would constitute strong evidence for a veridical trait-level relation. The scales that were administered directly before the religious belief measures differed across the four studies (see S1 Text for a breakdown) and their order within the mass testing survey was always randomized. As such, the likelihood that any association can be explained by an unexpected confound (which, in our view, is low in the first place) decreases with the increasing number of studies.

## Method

### Ethics statement

These studies were approved by a University of Waterloo Research Ethics Committee. Participants signed and received separate consent forms for the mass testing and analytic thinking surveys, which were all completed online. All participants indicated their willingness to consent via button press. Participants received course credit for the mass testing and analytic thinking surveys.

### Participants

Participants completed a series of reasoning problems and cognitive ability measures as part of a larger project on the everyday consequences of analytic thinking (see [2]). This includes, but is not limited to, religious belief. All participants were University of Waterloo students who signed up for a study on “thinking styles and reasoning” online through the participant pool. The only exclusion criteria was that participants had to have completed the department-wide mass-testing and pre-screen questionnaires prior to signing up for the thinking styles study (not every student in the participant pool completes one or both of these questionnaires). Participants were not permitted to sign up for the study more than once (i.e., someone who participated in Study 1 was not permitted to sign up for Studies 2–4 in the subsequent semesters). Demographic characteristics of the participants can be found in Table 1.

**Table 1:** Demographics for full participant samples (i.e., excluding those who failed the attention check) in Studies 1–4.

	Study 1	Study 2	Study 3	Study 4
*N*_females_	265	99	205	192
*N*_males_	107	50	73	75
*N*_total_	372	149	279^a^	267
Age (SD)	20.3 (3.9)	21.7 (4.9)	20.2 (3.9)	20.7 (5.2)

^a^ One participant did not indicate their gender.

#### Study 1

We had complete data for 381 participants. At the end of the analytic thinking survey (but not in the mass testing or pre-screen surveys), participants were given an attention check question. The same instruction check was used for all four studies, with some slight variations (see below). For this, they were presented with a list of common activities and, in the instruction box, it read: “Below is a list of leisure activities. If you are reading this, please choose the ‘other’ box below and write ‘I read the instructions’.” Nine participants failed the instruction check and were excluded from subsequent analysis. This left us with 372 participants (see Table 1). We also asked participants if they had seen the CRT before and 77 participants (20.7%) responded affirmatively. Their data were retained but analyses are reported with and without these participants. Study 1 was completed in the winter term of 2013. A subset of the data from Study 1, 3, and 4 were previously published in an investigation of CRT scoring strategies [15]. For this, participants who completed the analytic thinking survey were permitted to complete a second survey with analytic thinking disposition measures and this subset of participants are included in Pennycook et al. [15]. In the current studies, the full sample is included and additional data from thinking disposition questionnaires is reported in S2 Text. The results for the thinking disposition questionnaires parallel our performance-based findings.

#### Study 2

We had complete data for 158 participants. Nine participants failed the instruction check and were excluded from subsequent analysis. This left us with 149 participants (see Table 1), 20.7% (*N* = 31) of which responded affirmatively when asked if they have seen the CRT before. Study 2 was completed in the spring term of 2013.

#### Study 3

We had complete data for 406 participants. The attention check was made more difficult for Studies 3 and 4 by inserting an introductory screen prior to the instruction check that said: “For the final part of the study, we are interested in the types of things that you do in your spare time”. This had a large effect: 127 participants failed and were excluded from subsequent analysis. This left us with 279 participants (see Table 1), 21.5% (*N* = 60) of which responded affirmatively when asked if they have seen the CRT before. Study 3 was completed in the fall term of 2013. Components of this data set were also previously published in an investigation of analytic thinking and smartphone use [9].

#### Study 4

We had complete data for 398 participants. One hundred and thirty one participants failed the instruction check and were excluded from subsequent analysis. This left us with 267 participants (see Table 1), 25.1% (*N* = 67) of which responded affirmatively when asked if they have seen the CRT before. Study 4 was completed in the winter term of 2014.

### Materials

All items can be found in S3 Text. A breakdown of the materials for each study can be found in Table 2. Base-rate neglect problems, used in Studies 1 and 2, were replaced with a longer heuristics and biases battery in Studies 3 and 4.

**Table 2:** Materials for Studies 1–4. CRT = Cognitive Reflection Test.

		Study 1	Study 2	Study 3	Study 4
Religiosity	Religious Belief Scale	X	X	X	X
	God Type	X	X	X	X
	Religious Affiliation	X	X	X	X
Analytic Cognitive Style	CRT (original)	X	X	X	X
	CRT (additional)	X^a^		X	X
	Base-Rate Neglect	X	X		
	Heuristics/Biases			X	X
Cognitive Ability	Numeracy	X	X	X	X
	Wordsum	X	X	X	X

^a^ The additional CRT questions in Study 1 (*N* = 3) differed from those used in Studies 3 and 4 (*N* = 4; see S3 Text.

#### Religious beliefs

The religious belief scale from the mass testing survey included statements about eight conventional religious beliefs (Pennycook et al. [22], Study 2): heaven, hell, miracles, afterlife, angels, demons, soul, and the devil/Satan. Participants indicated their agreement/disagreement with the statements (where agreement meant that they held the belief in question) on the following 5-point scale: 1) I strongly disagree, 2) I disagree, 3) I don’t know, 4) I agree, 5) I strongly agree. The scale had good internal consistency: Cronbach’s α = 0.94 in each of the four studies and in the combined data set. Participants also indicated the ‘type’ of God that they believed in on a 7 point scale [4]: 1) A personal God, 2) God as an impersonal force, 3) A God who created everything, but does not intervene in human affairs [Deism], 4) Don’t know whether or not any Gods exist [Negative Agnostic], 5) Don’t know whether or not any Gods exist and no one else does either [Positive Agnostic], 6) I don’t believe in Gods of any sort [Negative Atheist], and 7) I believe that God does not exist [Positive Atheist]. To ease exposition, a theism measure was created by combining theists (options 1–3), agnostics (options 4 & 5), and atheists (options 6 & 7).

For the religious affiliation question in the pre-screen survey, participants were presented with a list of religious affiliations and asked to select the option that they most strongly identified with. The list included the following options: Agnostic, Atheist, Baha’i, Buddhist, Chinese Traditional, Christian, Christian (specifically Catholic), Christian (specifically Protestant), Hindu, Humanist, Jewish, Muslim, No religion, Sikh, Taoist, and Other/not listed. Across the entire data set (i.e., Studies 1–4 combined), 13.3% (*N* = 142) of the sample selected agnostic, 12.5% (*N* = 133) selected atheist, 16% (*N* = 171) of the sample chose ‘no religion’, 42% (*N* = 448) of the sample selected one of the three Christian options, 14.8% (*N* = 149) chose a non-Christian religious affiliation. The remaining 2.2% (*N* = 24) did not provide a response and were excluded from the affiliation analysis.

#### Analytic cognitive style

Multiple measures of analytic cognitive style were included across the four studies. Each measure is intended to cue an incorrect intuitive response that requires additional analytic processing to override. The original 3-item CRT [14] was included in each study. Additional CRT items were added for Studies 1, 3, and 4. Three items were added for Study 1 and four items were added for Studies 3 and 4 [17]. In Studies 1 and 2, we also included 6 incongruent base-rate neglect problems [22]. These problems were intermixed with 6 congruent and 6 neutral problems. The following is an incongruent problem [31]:

In a study 1000 people were tested. Among the participants there were 995 nurses and 5 doctors. Paul is a randomly chosen participant of this study. Paul is 34 years old. He lives in a beautiful home in a posh suburb. He is well spoken and very interested in politics. He invests a lot of time in his career. What is most likely?
(a) Paul is a nurse.
(b) Paul is a doctor.

Base-rate neglect refers to the propensity for individuals to underweight or ignore the base-rate information (i.e., 995 nurses/5 doctors) in lieu of the more intuitive stereotypical information (i.e., Paul more closely resembles the stereotype of a doctor than a nurse). Incongruent problems contain a conflict between base-rate and stereotype (as above) whereas both sources of information suggest the same response for congruent problems. Neutral problems do not contain stereotypes in the personality description. The proportion of base-rate responses for incongruent problems has been shown to correlate negatively with religious belief in prior work [4, 22]. Finally, in Studies 3 and 4, a 14-item battery of heuristics and biases problems was administered [9, 10, 16, 17]. The battery included problems such as the conjunction fallacy and the gambler’s fallacy.

#### Cognitive ability

Measures of cognitive ability were included as control variables in each of the four studies. For this, a 12-item verbal intelligence test (the “Wordsum” [32]) was administered in each study. Participants also completed a 3-item numeracy test [33] in Studies 1, 3, and 4. This was increased to a 5-item test in Study 2 [34].

### Procedure

#### Mass testing

The religious belief scale was administered in online mass testing surveys with a number of different scales. These scales differed across the four studies (see S1 Text for a breakdown), but their order within the mass testing survey was always randomized.

#### Pre-screen

The religious affiliation question always came after a set of demographic questions taken in an online pre-screen questionnaire.

#### Analytic thinking survey

Participants first completed the base-rate neglect/heuristics and biases problems (depending on which study, see Table 2), followed by numeracy and the CRT. The Wordsum was always administered last.

#### Order

Participants had to complete the mass testing and pre-screen surveys before they were eligible to sign up for the primary analytic thinking survey. The vast majority of participants completed the analytic thinking survey on a different day than the mass testing and/or pre-screen surveys. However, a relatively small proportion did complete the analytic thinking survey on the same day as the mass testing (8.5% of the sample) or pre-screen (3.9% of the sample) surveys. Nonetheless, there was nothing linking the analytic thinking survey to any individual measure within the mass testing or pre-screen batteries and the results are essentially identical for participants who completed the surveys on the same day (see S4 Text). We therefore retained the full sample in the following analyses.

## Results

Descriptive statistics for cognitive variables can be found in Table 3. Data for the original 3-item CRT are reported with (CRT^1^) and without (CRT^2^) participants who indicated seeing the measure before. An analytic cognitive style (ACS) measure was computed by taking the mean of the original CRT (including participants who had seen it before, CRT^1^), and, when appropriate, the extended CRT measure (CRT^3^), base-rate neglect, and heuristics/biases. A correlation matrix for all cognitive variables collapsing across study can be found in Table 4.

**Table 3:** Mean proportion correct and associated standard deviations and skewness (in brackets: SD /_Skew_) for cognitive variables.

	Study 1	Study 2	Study 3	Study 4	Combined
CRT^1^	.39 (.37 /_.42_)	.40 (.39 /_.41_)	.37 (.36 /_.46_)	.43 (.38 /_.28_)	.40 (.37 /_.40_)
CRT^2^	.38 (.36 /_.46_)	.40 (.39 /_.43_)	.36 (.36 /_.51_)	.42 (.39 /_.32_)	.39 (.37 /_.43_)
CRT^3^	.36 (.33 /_.53_)	-	.41 (.33 /_.35_)	.45 (.32 /_.26_)	.40 (.33 /_.38_)
Base-Rate Neglect	.35 (.36 /_.72_)	.38 (.34 /_.58_)	-	-	.36 (.35 /_.68_)
Heuristics/ Biases	-	-	.52 (.18 /_.44_)	.53 (.17 /_.19_)	.52 (.17 /_.32_)
ACS	.37 (.27 /_.50_)	.39 (.29 /_.33_)	.43 (.24 /_.56_)	.47 (.24 /_.34_)	.41 (.26 /_.38_)
Numeracy	.73 (.30 /_-.78_)	.80 (.24 /_-1.12_)	.73 (.28 /_-.78_)	.76 (.30 /_-1.01_)	.75 (.29 /_-.89_)
Wordsum	.59 (.18 /_-.06_)	.63 (.17 /_-.17_)	.60 (.17 /_.15_)	.62 (.18 /_-.12_)	.60 (.18 /_-.05_)

CRT = Cognitive Reflection Test

CRT^1^ = Accuracy on original 3-item CRT

CRT^2^ = Excludes participants who indicated seeing the CRT before

CRT^3^ = Accuracy on additional CRT problems; ACS = Analytic Cognitive Style (mean of CRT^1^, CRT^3^, Base-Rate Neglect, Heuristics/Biases).

**Table 4:** Correlations (*r*) among cognitive variables, collapsing across all four studies.

	1	2	3	4	5	6	7
1. CRT^1^	-						
2. CRT^2^	-	-					
3. CRT^3^	.57***_(918)_	.57***_(884)_	-				
4. Base-Rate Neglect	.26***_(520)_	.23***_(506)_	.23***_(372)_	-			
5. Heuristics/Biases	.51***_(546)_	.50***_(523)_	.48***_(546)_	-	-		
6. Numeracy	.42***_(1066)_	.42***_(1029)_	.38***_(918)_	.21***_(521)_	.41***_(546)_	-	
7. Wordsum	.38***_(1066)_	.37***_(1029)_	.35***_(918)_	.22***_(521)_	.41***_(546)_	.30***_(1067)_	-

***indicates *p* < .001.

CRT = Cognitive Reflection Test

CRT^1^ = Accuracy on original 3-item CRT

CRT^2^ = Excludes participants who indicated seeing the CRT before

CRT^3^ = Accuracy on additional CRT problems. *N* for each correlation listed in brackets.

Pearson product-moment correlations (*r*) between the religious belief scale (taken in the mass-testing survey) and performance on cognitive tests is reported in Table 5. Consistent with previous research, religious belief was significantly negatively correlated with every analytic cognitive style measure in each of the four studies and in the combined data set (*r*’s ranging from -.16 to -.26). This association was strongest for the composite ACS measure (*r*’s ranging from -.20 to -.29). The negative correlation between the composite ACS measure and religious belief in the combined data set (*N* = 1,065) was -.26, 95% confidence interval (CI) [-.32, -.20]. This is in the middle of the range reported in the three original studies [4, 12, 13] using the CRT (*r*’s ranging from -.18 to -.33), as would be expected if those studies were measuring a genuine correlation between religious belief and analytic cognitive style. An interesting additional observation is that excluding participants who had previously seen the CRT did not have an effect on the association with religious belief (both *r*’s = .22 in the combined data set).

**Table 5:** Correlations (*r*) between religious belief (taken in a mass-testing survey) and performance on cognitive tests.

	CRT^1^	CRT^2^	CRT^3^	Base-Rate Neglect	Heuristics/Biases	ACS	Numeracy	Wordsum
Study 1	-.26***_(372)_	-.24***_(361)_	-.17**_(372)_	-.23***_(372)_	-	-.29***_(372)_	-.13*_(372)_	-.17**_(372)_
Study 2	-.21*_(148)_	-.22**_(145)_	-	-.25**_(149)_	-	-.29***_(149)_	-.17*_(149)_	-.10 _(149)_
Study 3	-.17**_(277)_	-.16*_(267)_	-.16**_(277)_	-	-.16**_(277)_	-.20**_(277)_	-.08 _(277)_	-.10 _(277)_
Study 4	-.23***_(267)_	-.25***_(254)_	-.21**_(267)_	-	-.21**_(267)_	-.26***_(267)_	-.05 _(267)_	-.26***_(267)_
Combined	-.22***_(1064)_	-.22***_(1027)_	-.18***_(916)_	-.23***_(521)_	-.18***_(544)_	-.26***_(1065)_	-.11**_(1065)_	-.17***_(1065)_

***indicates *p* < .001

**indicates p < .01

*indicates p < .05.

CRT = Cognitive Reflection Test

CRT^1^ = Accuracy on original 3-item CRT

CRT^2^ = Excludes participants who indicated seeing the CRT before

CRT^3^ = Accuracy on additional CRT problems; ACS = Analytic Cognitive Style (mean of CRT^1^, CRT^3^, Base-Rate Neglect, Heuristics/Biases). *N* for each correlation listed in brackets.

The negative association between religious belief and cognitive ability was less consistent. Both numeracy and verbal intelligence (Wordsum) were significantly negatively correlated with religious belief in two of four studies and in the combined data set. To further investigate the associations between cognitive style, cognitive ability, and religious belief, we ran a regression analysis on the combined data set with religious belief as the dependent variable and ACS, numeracy, and verbal intelligence as predictors. Analytic cognitive style (*β* = -.24, *p* < .001, 95% CI [-.17, -.31]) and verbal intelligence (*β* = -.07, *p* = .026, 95% CI [-.01, -.14]) emerged as significant independent predictors whereas numeracy was not significant (*β* = .03, *p* = .459, 95% CI [-.04, .09]). These results did not change when dummy variables for Study were included in the regression model (Analytic cognitive style: *β* = -.24, *p* < .001, 95% CI [-.17, -.31]; verbal intelligence: *β* = -.07, *p* = .031, 95% CI [-.01, -.14]; numeracy: *β* = .03, *p* = .407, 95% CI [-.04, .09]).

**Fig 1:**
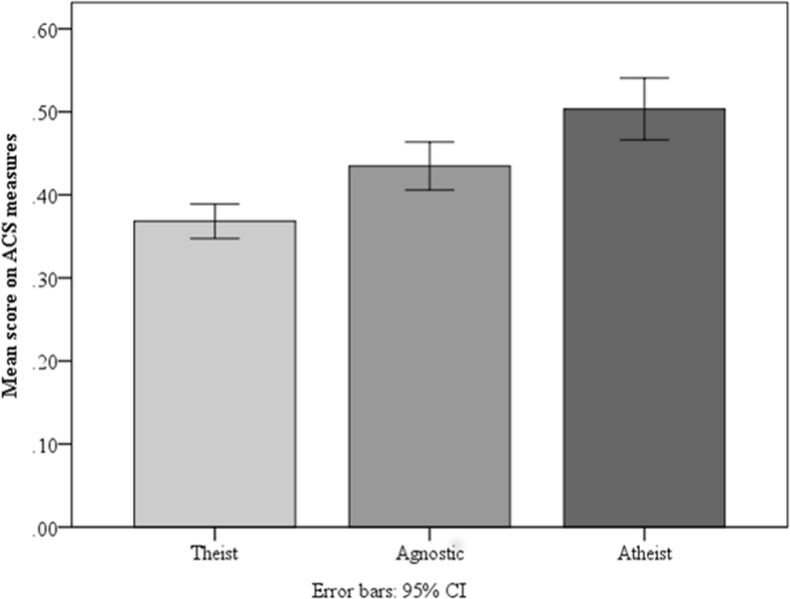
Mean accuracy on Analytic Cognitive Style (ACS) measures as a function of theism (taken in a mass testing survey). *N*’s = 570, 294, 200 (respectively).

To analyze the potential difference between theists and non-theists (agnostics and atheists), we ran individual one-way ANOVA’s for each cognitive measure. There was a main effect of theism in each case, all *F*’s > 4.28, *p*’s < .015, and for the composite ACS measure (see Fig 1), *F*(2, 1061) = 22.29, *MSE* = .065, *p* < .001, ƞ^2^ = .04, indicating increasing performance as level of theism decreased (i.e., from theist to agnostic to atheist). A post-hoc Tukey HSD test revealed significant differences between each of the groups, with agnostics scoring higher than theists (*p* = .001) and atheists scoring higher than agnostics (*p* = .01). Atheists scored 13.5% higher than theists on the ACS measures, *t*(768) = 6.40, *SE* = .02, *p* < .001, *d* = .52.

**Fig 2:**
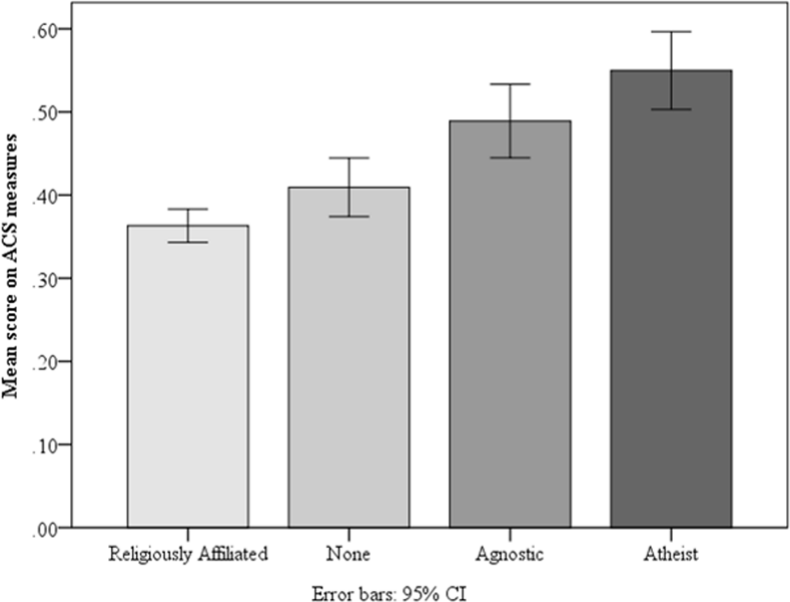
Mean accuracy on Analytic Cognitive Style (ACS) measures as a function of religious affiliation (taken in a pre-screen survey). *N*’s = 597, 171, 142, 133 (respectively).

We turn next to the religious affiliation question administered in the pre-screen surveys. For this, we created four groups using the self-reported affiliations. We focused on the combined data for this analysis to ensure that there was a sufficient sample size in each group. All participants who indicated a religious affiliation were put in a single “religiously affiliated” group (*N* = 597). Three non-religious groups were then created based on an increasing level of disaffiliation. Namely, those who indicated having no religion (“None”) were put in a group (*N* = 171) and self-identified agnostics (*N* = 142) and atheists (*N* = 133) were put into separate groups. Individual one-way ANOVA’s revealed that performance on every cognitive measure significantly differed across the four religious affiliation groups (all *F*’s > 5.84, all *p*’s ≤ .001). Focusing specifically on the composite ACS measure, there was a robust difference across groups, *F*(3, 1039) = 25.17, *MSE* = .064, *p* < .001, ƞ^2^ = .07. As is evident from Fig 2, atheists scored the highest, followed by agnostics, “nones”, and finally, the religiously affiliated. A post-hoc Tukey HSD test revealed that the religiously affiliated and “nones” were a homogeneous subset and the agnostics and atheists were a second homogeneous subset (*p*’s < .05). In other words, the religiously disaffiliated (atheists and agnostics) scored higher than the religiously affiliated and the religiously apathetic (“nones”). This is not a small effect: atheists scored 18.7% higher on the ACS composite than the religiously affiliated, *t*(728) = 7.69, *SE* = .02, *p* < .001, *d* = .72.

### Meta-Analysis

The results from these four studies provide strong support for the claim that atheists and agnostics are genuinely more reflective than are religious believers. Nonetheless, it may be the case that the *size* of the correlation between analytic thinking and religious belief reported in earlier studies has been inflated due to the CRT being presented before the religiosity questions. To investigate, we summarized the results of studies that have reported a zero-order correlation between at least one behavioral measure of analytic thinking (e.g., the CRT) and religiosity (Table 6). The order in which the analytic thinking/religiosity measures were administered for each of the studies is also identified in Table 6.

**Table 6:** Summary of studies reporting a correlation (*r*) between a behavioral measure of analytic thinking and religiosity (variously measured). Significant correlations are in bold.

Reference	Study	Analytic thinking measure	Religiosity measure	*r*	*N*
Shenhav et al.	1*	CRT (intuitive scoring)	God	**.18**^#^	882
(2012) [13]			Convinced of God’s existence	**.15**^a^	
			Immortal souls	**.14**	
			Belief change	**.19**	
	2*	CRT	God	**-.18**^#^	321
Pennycook et al.	1*	CRT	Religious belief scale	**-.33**^#^	181
(2012) [4]		Base-rate neglect		**-.19**	
	2*	CRT	Religious belief scale	**-.29**^#^	267
		Base-rate neglect		**-.31**	
Gervais &	1*	CRT	Intrinsic religiosity	**-.22**	179
Norenzayan			Intuitive religious belief	**-.15**	
(2012) [12]			Supernatural agents	**-.18**^#^	
Pennycook et al. (2013) [20]	1*	Belief bias syllogisms	Religious belief scale	**-.46**	91
Kahan (2013)^b^	1^§^	CRT	Importance of religion	**-.15**^#^	1750
[27]			Prayer frequency	**-.12**	
Razmyar &	1*	CRT	Overall religiosity	**-**.09	150
Reeve (2013)^c^			Overall spirituality	**-.19**	
[21]			Prayer frequency	**-.19**	
			Extrinsic religiosity	**-.20**	
			Intrinsic religiosity	**-.24**	
			Fundamentalism	-.10	
			Scriptural acceptance	**-.17**^#^	
Piazza & Sousa (2014) [35]	3*	CRT (intuitive scoring)	Overall religiosity	**.28**^#^	192
Pennycook et al.	1*	CRT	Religious belief scale	**-.23**^#^	505
(2014a) [7]		Base-rate neglect		**-.16**	
Pennycook et al.	1*	Base-rate neglect	Religious belief scale	**-.28**	78
(2014b) [22]	2^ŧ^	CRT	Religious belief scale	**-.26**^#^	198
		Base-rate neglect		**-.29**	200
	3^ŧ^	Base-rate neglect (rapid-response)	Religious belief scale	-.15	89
Browne et al.	1*	CRT	Strong faith	**-.11**^#^	1137
(2014)^d^ [30]			Spiritual thinking	**-.08**	
Byrd (2014)^e^ [26]	1^§^	CRT (intuitive scoring)	Theism	**.14**^#^	412
McCutcheon et	1^f^	CRT	Intrinsic religiosity	.04^#^	164
al. (2014) [36]		Belief bias syllogisms		-.02	
Baron et al. (2015) [37]	4*	CRT/ Belief bias syllogisms (combined)	God determines morality	**-.32**^#^	96
Gervais^g^ (2015)	1*	CRT	God	**-.10**^#^	787
[5]	2*	CRT	God	**-.09**^#^	596
Pennycook et al.	1*	CRT	Religious belief scale	**-.21**^#^	279
(2015) [10]		Heuristics & Biases battery		**-.20**	
	2*	Heuristics & Biases battery	Religious belief scale	**-.34**	187
Finley et al.	CRT	CRT	Intrinsic religiosity	**-.17**	410
(2015) [24]	First*		Intuitive religious belief	**-.23**	
			Supernatural agents	**-.19**^#^	
	Belief	CRT	Intrinsic religiosity	.04	410
	First^§^		Intuitive religious belief	< .01	
			Supernatural agents	-.03^#^	
Lindeman & Lipsanen (2016) [28]	1^§^	CRT	Religious belief scale	**-.22**^#^	3044
Jack et al. (in	1^§^	CRT	God	**-.15**^#^	236
press) [29]	2^§^	CRT	God	**-.25**^#^	233
	3*	CRT	God	**-.22**^#^	159
	4^§^	CRT	God	**-.24**^#^	527
	5^ŧ^	CRT	God	-.23^#^	69
	6*	CRT	God	**-.16**^#^	459
	8*	CRT	God	**-.17**^#^	371
Current study	1^ŧ^	CRT	Religious belief scale	**-.26**^#^	372
		Base-rate neglect		**-.23**	
	2^ŧ^	CRT	Religious belief scale	**-.21**^#^	148
		Base-rate neglect		**-.25**	149
	3^ŧ^	CRT	Religious belief scale	**-.17**^#^	277
		Heuristics/biases		**-.16**	
	4^ŧ^	CRT	Religious belief scale	**-.23**^#^	267
		Heuristics/biases		**-.21**	

^a^ Value is a point biserial correlation coefficient (dichotomous variable).

^b^ These values were computed by the present authors using Kahan’s (2013) [27] data, which were available online through the Society of Judgment and Decision Making website (http://journal.sjdm.org/vol8.4.html).

^c^ Some of these measures of religiosity relate to aspects of religious practice and commitment and not religious belief (see [11]).

^d^ The CRT was administered via phone interview in this study and performance was exceptionally low. This may explain the attenuated correlations.

^e^ This analysis excludes participants who had previous knowledge of the CRT. Around half of the sample includes philosophers either with a PhD or who were in a PhD program at the time of the study. Participants in this study were given the CRT before the theism measure, but with a personality task in-between.

^f^ The measures were completed in a paper-and-pencil study and the order of the pages was varied (no order analyses were reported).

^g^ These values were computed by the present authors using Gervais’ (2015) [5] data, which were available online through the author’s website (http://willgervais.com/journal-articles/). Participants with missing data for any CRT item were removed from analysis.

* Indicates that the religious belief measure was administered after the analytic thinking measure.

^§^ Indicates that the religious belief measure was administered before the analytic thinking measure.

^ŧ^ Indicates that the religious belief measure was administered in a separate session as the analytic thinking measure.

^#^ Indicates that the correlation was included in the meta-analysis.

Note: This table does not include correlations between religious belief and self-report measures of analytic thinking disposition (e.g., [38]).

As a procedure for identifying candidate studies, the primary author manually searched each article that cited any of the three original CRT/religiosity studies [4, 12, 13] via *Google Scholar*. Thirty-five studies were identified, including Finley et al.’s [24] two conditions (which we treat as two studies for the purpose of this analysis), the four studies from the present paper, an unpublished study from a Master’s thesis [26], and 8 studies (across 2 articles, [28, 29]) that were published after Finley et al. Thirty-one of these studies found a statistically significant negative association between analytic thinking and religious belief, and four did not. However, two of these non-significant results (*r*’s = -.15 and -.23 from [22] and [29], respectively) were within the range of previous work but had relatively small samples. The only two strong exceptions are from Finley et al. [24] and McCutcheon et al. [36]. Arguably, three large-sample studies in which the correlation between CRT performance and religiosity was significant, but quite modest could also be considered partial exceptions [5, 30]. However, in all three cases there were reasons why the correlation may have been attenuated. In the case of Browne et al. [30] (*r*’s = -.08 and -.11), the CRT was administered in a telephone interview and, likely as a consequence, their participants did particularly poorly (*Mean* accuracy = 0.46/3) thereby restricting the range. Similarly, Gervais’ [5] samples (*r*’s = -.09 and -.10) scored particularly low on the CRT (*Means* = 0.69 and 0.72 in Studies 1 and 2, respectively). Moreover, Gervais sampled from undergraduate students at the University of Kentucky who were highly religious (*Mean* belief in God was 5.88/7 in Study 1 and 76.7/100 in Study 2), indicating that the attenuated correlations may be the result of the restricted range for both the CRT and religious belief measure. We re-analyzed Gervais’ data (which is available online; see Table 6 notes) and found that self-identified agnostics/atheists (*N* = 69) scored almost 20% higher on the CRT (*Mean* = 1.20, *SD* = 1.17) than did the religiously affiliated (*N* = 517, *Mean* = 0.64, *SD* = 0.99), *t*(584) = 4.34, *SE* = .13, *p* < .001, *d* = .52. This is similar to the religious affiliation results from our four new studies.

We used meta-analysis to estimate the overall effect size of the association between religiosity and CRT scores. We focus specifically on the CRT as it is the most common measure across these studies and was the measure that Finley et al. employed. For the studies that used more than one religiosity measure, we chose the measure that was most closely related to religious belief (e.g., God, supernatural agents, faith). This is because the theoretical association between analytic thinking and religiosity pertains specifically to supernatural beliefs and not necessarily religious participation or practice [11]. Finally, three studies used an “intuitive scoring” approach for the CRT (see Table 6) in which performance was scored according to the number of intuitive (modal) responses instead of the number of correct responses. Given the strong correlation between the outcomes of these scoring techniques (e.g., *r* = -.85 in [15]), we simply reversed the sign of the correlation in the meta-analysis for the three relevant cases.

We converted *r* scores into Fisher’s z-scores to estimate uncertainty in effect sizes, and back-transformed Fisher’s z-scores to *r* scores for interpretation [39]. We conducted a random effects meta-analysis using Comprehensive Meta-Analysis version 3.3.070 [40, 41] and examined the data for publication bias using a funnel plot and Egger’s regression test for funnel plot asymmetry [42].

**Fig 3:**
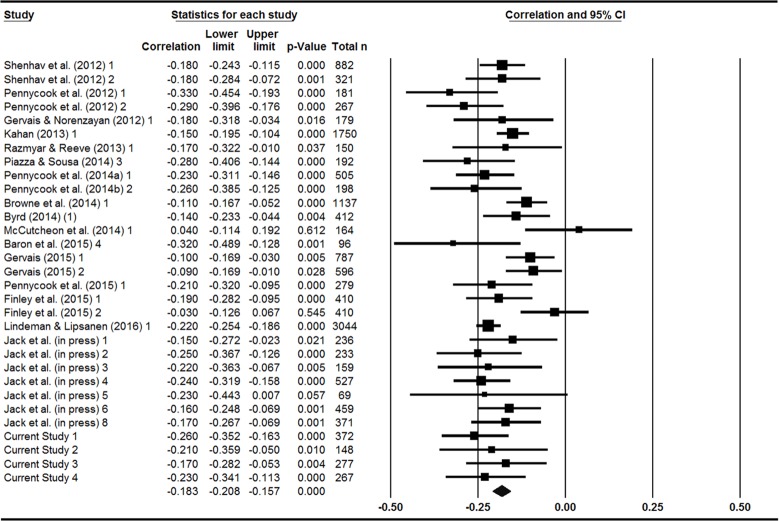
Forest plot of random effect meta-analysis showing effect sizes (*r*) for the association between religious belief scales and performance on the CRT.

Fig 3 shows a forest plot for the meta-analysis. The analysis indicates that there is a negative association between CRT score and religious belief, *r* = -.183 (95% CI [-.208, -.157], *N* = 15,078, *k* = 31). It is noteworthy that this overall effect size is the same as was reported in the very first study on the topic by Shenhav, Rand, and Greene [13]. Nonetheless, there was significant heterogeneity among the studies (*Q* = 64.16, *p* < .001, *I*^2^ = 53.24%), which indicates that the precise magnitude of the overall effect size should be interpreted with some degree of caution. This may (at least partially) be the result of the large differences in religiosity measures along with the restricted range of CRT scores in some of the studies (as discussed above).

Interestingly, a subgroup analysis of the 7 studies in which the religiosity measure was administered prior to the CRT (including Finley et al.’s belief first condition, see Table 6) produced a significant overall effect, *r* = -.174 (95% CI [-.227, -.120], *N* = 6,612, *k* = 7). The same was true for the 17 studies in which the CRT was administered first, *r* = -.183 (95% CI [-.216, -.150], *N* = 6,971, *k* = 17), and the 7 studies in which religiosity was measured in a separate session, *r* = -.228 (95% CI [-.290, -.163], *N* = 1,331, *k* = 7). Given the small number of studies in these subgroup analyses, the precise magnitude of the correlations should not be considered reliable nor should the small differences between them be strongly interpreted. The purpose of these subgroup analyses is to demonstrate that the negative association between CRT performance and religious belief is far more robust than implied by Finley et al. This may be because the majority of studies that measured religiosity in the same session as the CRT (regardless of order, excepting Finley et al.) included additional seemingly unrelated intervening measures, thereby masking the goal of the study.

**Fig 4:**
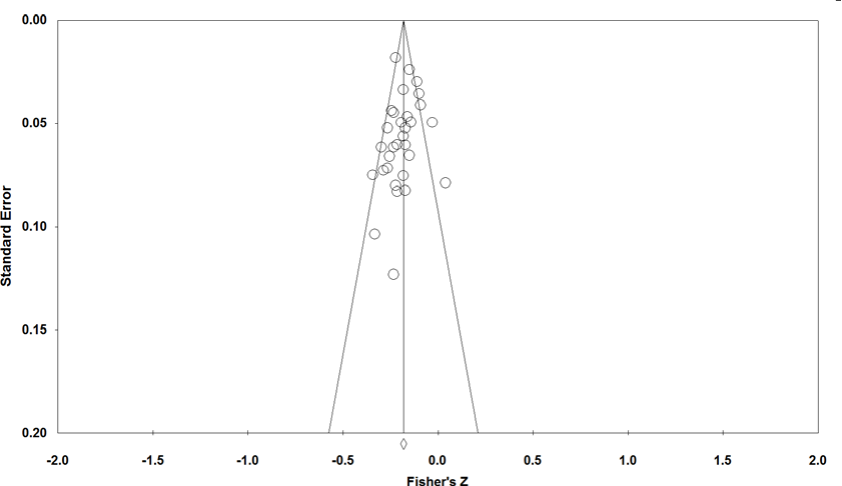
Funnel plot of standard error by Fisher’s Z.

Since our meta-analysis focused on published data (along with one Master’s thesis), it is possible that the overall effect size is inflated by publication bias. Since the correlation between CRT performance and religiosity is negative, publication bias should emerge as a gap in the bottom right region of a funnel plot (see Fig 4). Visual inspection reveals that the funnel plot for this meta-analysis is relatively symmetric. Consistent with this, Egger’s regression test for funnel plot asymmetry was not statistically significant (*t* = 0.74, *SE* = 0.61, *p* = .234), indicating little evidence for publication bias in this collection of studies.

## General Discussion

A negative association between analytic thinking and religious belief has been reported in several studies [11]. We replicated this association in four studies, totaling over 1,000 participants. Crucially, this association was evident despite the fact that religiosity and analytic thinking were measured in separate sessions. These results contradict Finley et al.’s [24] hypothesis that the association between religiosity and analytic thinking requires participants to be put in an analytic mindset when reporting their religiosity. In each of these four studies, our religious belief scale was administered in a mass testing survey along with a variety of scales (presented in a random order for each participant; see S1 Text). Analytic thinking was also predictive of categorical theism and religious affiliation variables (administered in mass testing and pre-screen surveys, respectively).

We also performed a meta-analysis to quantitatively examine the robustness of the relationship between analytic thinking and religious belief. This meta-analysis revealed evidence for a negative association between performance on the Cognitive Reflection Test and religious belief when considering all published studies (*r* = -.18). Although this effect size is small, it is similar to effect sizes for other cognitive factors often considered important in the development of religious belief. To take a few examples, researchers have found statistically significant associations between religious or theistic belief and mentalizing (*r* = .10, [43]), teleological thinking (*r* = .12 to .20, [43]), anthropomorphism (*r* = .05 to.10, [43]), empathizing (*r* = .21, [44]), ontological confusions (*r* = .22 to .35, [10, 44]), and dualism (*r* = .41, [43]). Moreover, although the overall correlation is relatively small, self-identified atheists scored 18.7% higher than religiously affiliated individuals on a composite measure of analytic thinking in the combined analysis of our four new studies (*d* = .72). This analysis was paralleled in Gervais’ [5] Study 2 (as reported above, see also [45]).

The results summarized in Table 6 seem to indicate that full religious belief scales (as used here, see also [4, 7, 10, 20, 22, 28]) may be preferable to single-item or more general religiosity measures. Moreover, the results of the four new studies reported here indicate that composite measures of analytic thinking may prove to be better predictors of religious belief than the 3-item Cognitive Reflection Test, which is by far the most common measure used in this literature. Finally, given the robust differences between self-reported atheists/agnostics and religious believers, samples that do not contain a sufficient number of religious disbelievers may not produce strong negative associations between religious belief and performance on analytic thinking measures. This is an area that requires further exploration.

Finley et al. framed their discussion in terms of explaining why religious belief might correlate with analytic thinking when the CRT is administered prior to questions about religiosity. Our results indicate that a more appropriate question might be why there was no correlation when religiosity was measured first by Finley et al. In our initial investigation of the relationship between analytic thinking and religiosity [4], we deliberately measured religiosity last due to concerns that demand characteristics might influence how participants respond to other questions. In particular, we suspect that the object of the study might become transparent to some participants when religiosity and analytic thinking are measured in proximity to each other and without any cover story. Given the claim that the CRT and related measures are sensitive to the disposition to think analytically [18], we reasoned that religious beliefs (which are presumably more stable) should be administered second (although there is evidence that the strength of particular religious beliefs is affected by analytic primes [12, 13]). Put another way, Finley et al.’s failure to replicate might have occurred because participants correctly guessed that the study had something to do with religion by the time they were given the CRT. This may have motivated religious participants to perform well on the CRT, thereby diminishing the influence of analytic thinking disposition (i.e., the motivation to think when not otherwise compelled to do so). This conjecture is supported by the fact that prior studies have shown a negative correlation between CRT performance and religious belief even when the CRT came at the end of the study (see Table 6). For example, Byrd [26] found a correlation between the CRT and theism when participants were given a personality test after the religious belief question but before the CRT, which may have masked the goal of the study. It should nonetheless be noted that Finley et al. did not find a significant difference in CRT performance between order conditions. If demand characteristics explain Finley et al.’s failure to replicate, the mechanisms may be more complicated than outlined here. For example, some religious participants may be motivated by the demand characteristic whereas others may simply give up on the task. Future research is needed to come to any definitive conclusions.

### Conclusion

Finley et al. [24] hypothesised that there should only be a correlation between analytic thinking and religious belief when participants are put in an analytic thinking mindset prior to reporting their level of religious belief. Across four new studies, we found that this is not the case. There was a consistent negative association between performance on analytic thinking measures and religious belief even when the two measures were administered in separate surveys. Moreover, there was an association between analytic thinking and categorical religious belief variables: Self-identified atheists and agnostics scored higher on various measures of analytic cognitive style than did religious believers. A summary and meta-analysis of prior work indicates that Finley et al.’s failed replication is one of only two notable exceptions across 35 studies with over 15,000 participants. These results reinforce prior work and indicate that, contra Finley et al., there is a genuine association between analytic thinking and religious disbelief.

## Supporting Information

S1 Text Mass testing. (XLSX)

S2 Text Results for thinking disposition scales. (DOCX)

S3 Text Materials. (DOCX)

S4 Text Analysis of same-day/different-day participants. (DOCX)
