# Correction: Atheists and Agnostics Are More Reflective than Religious Believers: Four Empirical Studies and a Meta-Analysis

**DOI:** 10.1371/journal.pone.0176586

**Published:** 2017-04-21

**Authors:** Gordon Pennycook, Robert M. Ross, Derek J. Koehler, Jonathan A. Fugelsang

There is an error in one of the correlations (*r*) between Cognitive Reflection Test (CRT) performance and belief in God in Table 6, Fig 3 and Fig 4. The correct correlation, from Gervais [5], is -.16. Please see the corrected Table 6, Fig 3 and Fig 4 below.

**Table 6:** Summary of studies reporting a correlation (*r*) between a behavioral measure of analytic thinking and religiosity (variously measured). Significant correlations are in bold.

Reference	Study	Analytic thinking measure	Religiosity measure	*r*	*N*
Shenhav et al.	1*	CRT (intuitive scoring)	God	**.18**^#^	882
(2012) [13]		w	Convinced of God’s existence	**.15**^a^	
			Immortal souls	**.14**	
			Belief change	**.19**	
	2*	CRT	God	**-.18**^#^	321
Pennycook et al.	1*	CRT	Religious belief scale	**-.33**^#^	181
(2012) [4]		Base-rate neglect		**-.19**	
	2*	CRT	Religious belief scale	**-.29**^#^	267
		Base-rate neglect		**-.31**	
Gervais &	1*	CRT	Intrinsic religiosity	**-.22**	179
Norenzayan			Intuitive religious belief	**-.15**	
(2012) [12]			Supernatural agents	**-.18**^#^	
Pennycook et al. (2013) [20]	1*	Belief bias syllogisms	Religious belief scale	**-.46**	91
Kahan (2013)^b^	1^§^	CRT	Importance of religion	**-.15**^#^	1750
[27]			Prayer frequency	**-.12**	
Razmyar &	1*	CRT	Overall religiosity	**-**.09	150
Reeve (2013)^c^			Overall spirituality	**-.19**	
[21]			Prayer frequency	**-.19**	
			Extrinsic religiosity	**-.20**	
			Intrinsic religiosity	**-.24**	
			Fundamentalism	-.10	
			Scriptural acceptance	**-.17**^#^	
Piazza & Sousa (2014) [35]	3*	CRT (intuitive scoring)	Overall religiosity	**.28**^#^	192
Pennycook et al.	1*	CRT	Religious belief scale	**-.23**^#^	505
(2014a) [7]		Base-rate neglect		**-.16**	
Pennycook et al.	1*	Base-rate neglect	Religious belief scale	**-.28**	78
(2014b) [22]	2^ŧ^	CRT	Religious belief scale	**-.26**^#^	198
		Base-rate neglect		**-.29**	200
	3^ŧ^	Base-rate neglect (rapid-response)	Religious belief scale	-.15	89
Browne et al.	1*	CRT	Strong faith	**-.11**^#^	1137
(2014)^d^ [30]			Spiritual thinking	**-.08**	
Byrd (2014)^e^ [26]	1^§^	CRT (intuitive scoring)	Theism	**.14**^#^	412
McCutcheon et	1^f^	CRT	Intrinsic religiosity	.04^#^	164
al. (2014) [36]		Belief bias syllogisms		-.02	
Baron et al. (2015) [37]	4*	CRT/ Belief bias syllogisms (combined)	God determines morality	**-.32**^#^	96
Gervais^g^ (2015)	1*	CRT	God	**-.10**^#^	787
[5]	2*	CRT	God	**-.16**^#^	596
Pennycook et al.	1*	CRT	Religious belief scale	**-.21**^#^	279
(2015) [10]		Heuristics & Biases battery		**-.20**	
	2*	Heuristics & Biases battery	Religious belief scale	**-.34**	187
Finley et al.	CRT	CRT	Intrinsic religiosity	**-.17**	410
(2015) [24]	First*		Intuitive religious belief	**-.23**	
			Supernatural agents	**-.19**^#^	
	Belief	CRT	Intrinsic religiosity	.04	410
	First^§^		Intuitive religious belief	< .01	
			Supernatural agents	-.03^#^	
Lindeman & Lipsanen (2016) [28]	1^§^	CRT	Religious belief scale	**-.22**^#^	3044
Jack et al. (in	1^§^	CRT	God	**-.15**^#^	236
press) [29]	2^§^	CRT	God	**-.25**^#^	233
	3*	CRT	God	**-.22**^#^	159
	4^§^	CRT	God	**-.24**^#^	527
	5^ŧ^	CRT	God	-.23^#^	69
	6*	CRT	God	**-.16**^#^	459
	8*	CRT	God	**-.17**^#^	371
Current study	1^ŧ^	CRT	Religious belief scale	**-.26**^#^	372
		Base-rate neglect		**-.23**	
	2^ŧ^	CRT	Religious belief scale	**-.21**^#^	148
		Base-rate neglect		**-.25**	149
	3^ŧ^	CRT	Religious belief scale	**-.17**^#^	277
		Heuristics/biases		**-.16**	
	4^ŧ^	CRT	Religious belief scale	**-.23**^#^	267
		Heuristics/biases		**-.21**	

^a^ Value is a point biserial correlation coefficient (dichotomous variable).

^b^ These values were computed by the present authors using Kahan’s (2013) [27] data, which were available online through the Society of Judgment and Decision Making website (http://journal.sjdm.org/vol8.4.html).

^c^ Some of these measures of religiosity relate to aspects of religious practice and commitment and not religious belief (see [11]).

^d^ The CRT was administered via phone interview in this study and performance was exceptionally low. This may explain the attenuated correlations.

^e^ This analysis excludes participants who had previous knowledge of the CRT. Around half of the sample includes philosophers either with a PhD or who were in a PhD program at the time of the study. Participants in this study were given the CRT before the theism measure, but with a personality task in-between.

^f ^The measures were completed in a paper-and-pencil study and the order of the pages was varied (no order analyses were reported).

^g ^These values were computed by the present authors using Gervais’ (2015) [5] data, which were available online through the author’s website (http://willgervais.com/journal-articles/). Participants with missing data for any CRT item were removed from analysis.

* Indicates that the religious belief measure was administered after the analytic thinking measure.

^§^ Indicates that the religious belief measure was administered before the analytic thinking measure.

^ŧ^ Indicates that the religious belief measure was administered in a separate session as the analytic thinking measure.

^#^ Indicates that the correlation was included in the meta-analysis.

Note: This table does not include correlations between religious belief and self-report measures of analytic thinking disposition (e.g., [38]).

**Fig 3:**
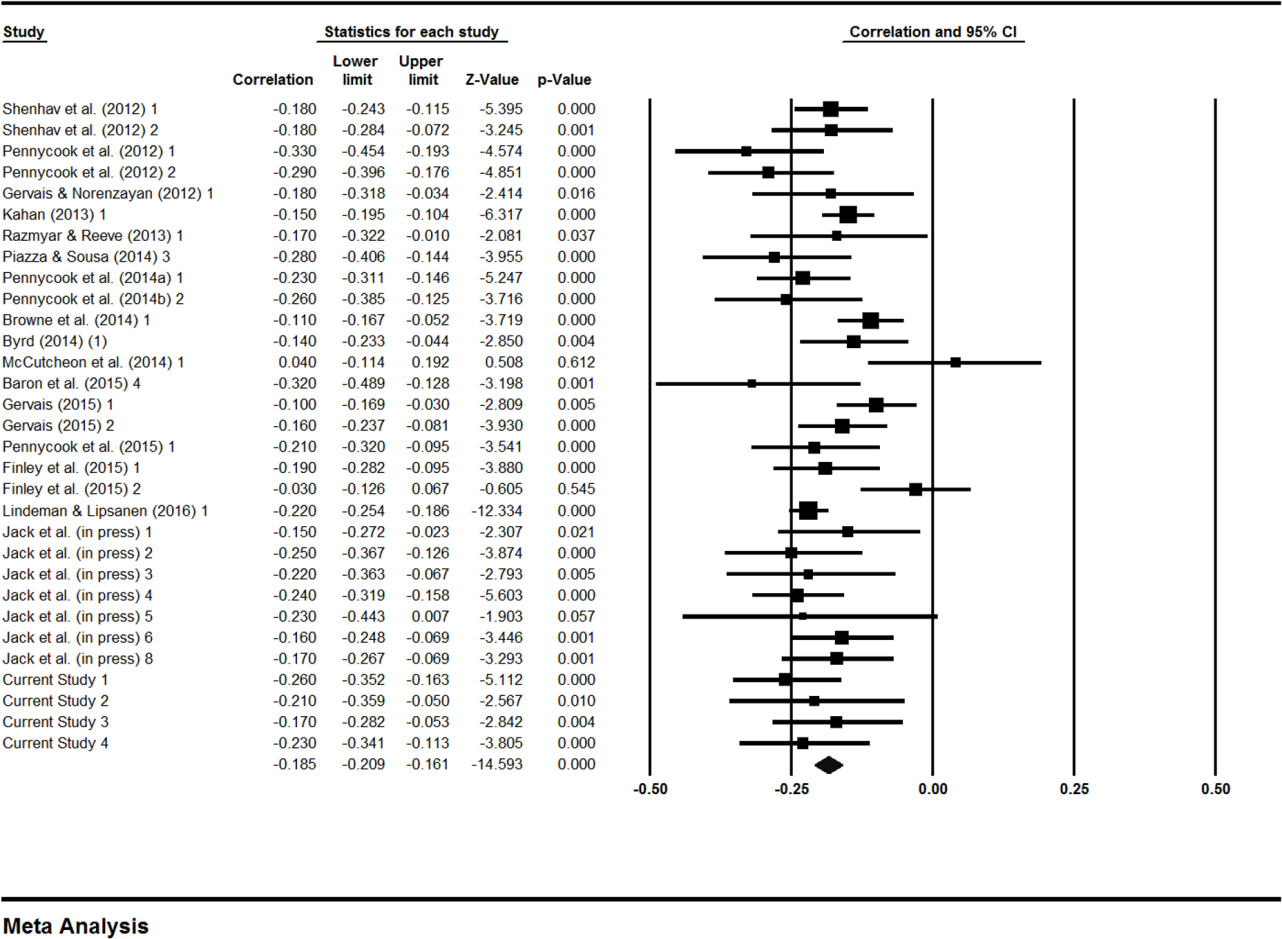
Forest plot of random effect meta-analysis showing effect sizes (*r*) for the association between religious belief scales and performance on the CRT.

**Fig 4:**
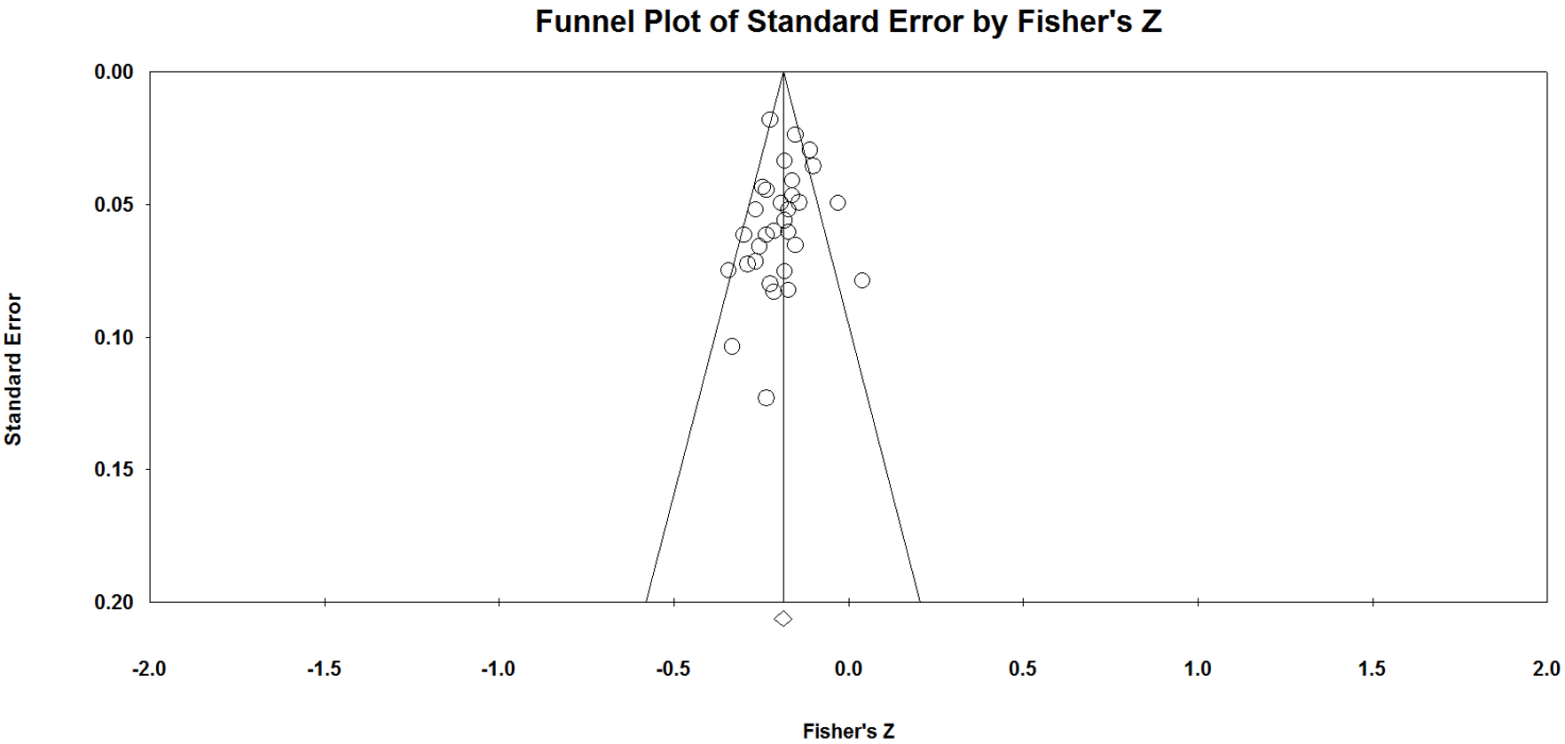
Funnel plot of standard error by Fisher’s Z.
