# Supplementary material for: Atheists and Agnostics Are More Reflective than Religious Believers: Four Empirical Studies and a Meta-Analysis
Source: PLoS One. 2016 Apr 7;11(4):e0153039. doi: 10.1371/journal.pone.0153039 (PMC4824409; doi:10.1371/journal.pone.0153039)
Supplement: S2 Text — (DOCX) [file pone.0153039.s002.docx]

**Results for Thinking Disposition Scales**

Participants who completed the battery of cognitive tests described in the manuscript were also permitted to sign-up for a study that consisted of a series of thinking disposition questionnaires. These data were published in an investigation of CRT scoring techniques by Pennycook et al. (2015). To construct the following data set, we took the data from Pennycook et al. (2015) and imported the religiosity measures discussed in the manuscript. What follows is a combination of results for Studies 1, 3, and 4 (as discussed in the manuscript).

**Method**

*Participants*

We had complete data for 436 participants (*Mean*_age_ = 20.6, *SD*_age_ = 4.6; 297 females, 138 males, 1 participant did not indicate gender).

*Materials*

Participants completed three thinking disposition measures. From Pennycook et al. (2015): “We used Pacini and Epstein’s (1999) Rational–Experiential Inventory, which included a 20-item Need for Cognition (NFC) scale and a 20-item Faith in Intuition scale (FI)… Participants were given questions such as “reasoning things out carefully is not one of my strong points” (NFC, reverse scored) and “I like to rely on my intuitive impressions” (FI). They were asked to respond using a 5-point scale, from 1 (Definitely not true of myself) to 5 (Definitely true of myself).” Participants also complete Stanovich and West’s (2006) 41-item Actively Open-Minded Thinking scale (AOT). Participants were given questions such as “Beliefs should always be revised in response to new information or evidence.” All three scales had good internal consistency: Cronbach’s alpha = .87 (NFC), .88 (FI), .89 (AOT). Religiosity measures were as described in the manuscript.

**Results**

**Table S2.1.** Correlations (*r*) between thinking disposition scales and religious belief. NFC = Need for Cognition; FI = Faith in Intuition; AOT = Actively Open-Minded Thinking. *N* = 432.

|  | 1 | 2 | 3 | 4 |
| --- | --- | --- | --- | --- |
| 1. Religious Belief | - |  |  |  |
| 2. NFC | -.22^***^ | - |  |  |
| 3. FI | .18^***^ | -.07 | - |  |
| 4. AOT | -.45^***^ | .35^***^ | -.11^*^ | - |

***indicates *p* < .001, **indicates p < .01, *indicates p < .05.

As is evident from Table S2.1, religious belief correlated negatively with the analytic thinking disposition measures (AOT & NFC) and positively with the intuitive thinking disposition measure (FI). This correlation was stronger for the AOT than it was for the NFC or FI according to a William’s test, *t*’s > 4.7, *p*’s < .001, which is consistent with previous research (Svedholm & Lindeman, 2012).

**References**

Pacini, R., & Epstein, S. (1999). The relation of rational and experiential information processing styles to personality, basic beliefs, and the ratio-bias phenomenon. *Journal of Personality and Social Psychology, 76*, 972–987.

Pennycook, G., Cheyne, J.A., Koehler, D.J. & Fugelsang, J.A. (2015). Is the cognitive reflection test a measure of reflection and intuition? *Behavior Research Methods*. doi 10.3758/s13428-015-0576-1.

Stanovich, K. E., & West, R. F. (2006). Natural myside bias is independent of cognitive ability. *Thinking & Reasoning, 13*, 225–247.

Svedholm, A. M., & Lindeman, M. (2013). The separate roles of the reflective mind and involuntary inhibitory control in gatekeeping paranormal beliefs and the underlying intuitive confusions. *British Journal of Psychology, 3*, 303-319.
