# Supplementary material for: Atheists and Agnostics Are More Reflective than Religious Believers: Four Empirical Studies and a Meta-Analysis
Source: PLoS One. 2016 Apr 7;11(4):e0153039. doi: 10.1371/journal.pone.0153039 (PMC4824409; doi:10.1371/journal.pone.0153039)
Supplement: S3 Text — (DOCX) [file pone.0153039.s003.docx]

**Religiosity Measures**

**Religious belief scale**

|  | I strongly disagree (1) | I disagree (2) | I don't know (3) | I agree (4) | I strongly agree (5) |
| --- | --- | --- | --- | --- | --- |
| There is a life after death. (1) |  |  |  |  |  |
| There is a heaven where people who have led good lives are eternally rewarded. (2) |  |  |  |  |  |
| There is a hell where people who have led bad lives and die without being sorry are eternally punished. (3) |  |  |  |  |  |
| Religious miracles occur. (4) |  |  |  |  |  |
| Angels are active in the world. (5) |  |  |  |  |  |
| Demons are active in the world. (6) |  |  |  |  |  |
| People have an immaterial soul, a part of themselves that is beyond their merely physiological and physical properties. (7) |  |  |  |  |  |
| There is a devil/Satan. (8) |  |  |  |  |  |

**Theism Scale**

What sort of God, if any, do you believe in?

- A personal God (1)
- God as an impersonal force (2)
- A God who created everything, but does not intervene in human affairs (3)
- Don't know whether or not any Gods exist (4)
- Don't know whether any Gods exist, and no one else does either (5)
- I don't believe in Gods of any sort (6)
- I believe that God does not exist (7)

[Note: Theist = 1-3; Agnostic = 4 & 5; Atheist = 6 & 7]

**Religious Affiliation Scale**

With which of the following do you identify?

- Agnostic
- Atheist
- Baha'i
- Buddhist
- Chinese Traditional
- Christian
- Christian (specifically Catholic)
- Christian (specifically Protestant)
- Hindu
- Humanist
- Jewish
- Muslim
- No religion
- Other not listed
- Sikh
- Taoist

**Analytic thinking measures**

Correct responses are bolded (unless otherwise stated).

**Cognitive Reflection Test (original)**

Source: Frederick (2005)

1. A bat and a ball cost $1.10 in total. The bat costs $1.00 more than the ball. How much does the ball cost?

____ cents **(5)**

1. If it takes 5 machines 5 minutes to make 5 widgets, how long would it take 100 machines to make 100 widgets?

____ minutes **(5)**

1. In a lake, there is a patch of lily pads. Every day, the patch doubles in size. If it takes 48 days for the patch to cover the entire lake, how long would it take for the patch to cover half of the lake?

____ days **(47)**

**Cognitive Reflection Test (additional)**

Source: Toplak, West, & Stanovich (2014)

1) If John can drink one barrel of water in 6 days, and Mary can drink one barrel of water in 12 days, how long would it take them to drink one barrel of water together? _____ days **(4)**

2) Jerry received both the 15th highest and the 15th lowest mark in the class. How many students are in the class? ______ students **(29)**

3) A man buys a pig for $60, sells it for $70, buys it back for $80, and sells it finally for $90. How much has he made? _____ dollars **(20)**

4) Simon decided to invest $8,000 in the stock market one day early in 2008. Six months after he invested, on July 17, the stocks he had purchased were down 50%. Fortunately for Simon, from July 17 to October 17, the stocks he had purchased went up 75%. At this point, Simon has: a. broken even in the stock market, b. is ahead of where he began, c. has lost money **(c)**

[Note: Study 1 was completed before we had access to the scale from Toplak et al. (2014). However, the first two items (which had been shared prior) were used along with a third item (below). **Items 3 & 4 were not included in Study 1.]**

3) A white cube is painted black on the outside. If it were cut into 27 identical smaller cubes, how many would have exactly two black sides? _____ cubes **(12) *only Study 1***

**Base-rate neglect problems**

Source: De Neys & Glumicic (2008)

Note: Only incongruent base-rate problems are of theoretical interest. Congruent and neutral problems were intermixed with incongruent problems in a fix random order, but not analyzed in the manuscript. They can be found in De Neys & Glumicic (2008).

*Incongruent*

| 1 | In a study 1000 people were tested. Among the participants there were 5 engineers and 995 lawyers. Jack is a randomly chosen participant of this study.  Jack is 36 years old. He is not married and is somewhat introverted. He likes to spend his free time reading science fiction and writing computer programs.  What is most likely?   - Jack is an engineer - **Jack is a lawyer** |
| --- | --- |
| 2 | In a study 1000 people were tested. Among the participants there were 995 fifty-year olds and 5 sixteen-year olds. Ellen is a randomly chosen participant of this study.  Ellen likes to listen to hip hop and rap music. She enjoys wearing tight shirts and jeans. She's fond of dancing and has a small nose piercing.  What is most likely?   - **Ellen is fifty years old** - Ellen is sixteen years old |
| 3 | In a study 1000 people were tested. Among the participants there were 996 women and 4 men. Jessie is a randomly chosen participant of the study.  Jessie is 23 years old and is finishing a degree in engineering. On Friday nights, Jessie likes to go out with friends and listen to loud music and drink beer.  What is most likely?   - **Jessie is a woman** - Jessie is a man |
| 4 | In a study 1000 people were tested. Among the participants there were 3 who live in a condo and 997 who live in a farmhouse. Kurt is a randomly chosen participant of this study.  Kurt works on Wall Street and is single. He works long hours and wears Armani suits to work. He likes wearing sunglasses.  What is most likely?   - Kurt lives in a condo - **Kurt lives in a farmhouse** |
| 5 | In a study 1000 people were tested. Among the participants there were 4 whose favorite series is Star Trek and 996 whose favorite series is Glee. Jeremy is a randomly chosen participant of this study.  Jeremy is 26 and is doing graduate studies in physics. He stays at home most of the time and likes to play video-games.  What is most likely?   - Jeremy's favorite series is Star Trek - **Jeremy's favorite series is Glee** |
| 6 | In a study 1000 people were tested. Among the participants there were 997 nurses and 3 doctors. Paul is a randomly chosen participant of this study.  Paul is 34 years old. He lives in a beautiful home in a posh suburb. He is well spoken and very interested in politics. He invests a lot of time in his career.  What is most likely?   - **Paul is a nurse** - Paul is a doctor |

**Heuristics and Biases Inventory**

Source: Toplak, West, & Stanovich (2011)

1. *Causal Base-Rate*

The Caldwells had long ago decided that when it was time to replace their car they would get what they called “one of those solid, safety-conscious, built-to-last Swedish cars”, either a Volvo or a Saab.

As luck would have it, their old car gave up the ghost on the last day of the closeout sale for the model year both for the Volvo and for the Saab. The model year was changing for both cars and the dollar had recently dropped substantially against European currencies; therefore, if they waited to buy either a Volvo or a Saab, it would cost them substantially more, about $2500.

They quickly got out their Consumer Reports where they found that the consensus of the experts was that both cars were very sound mechanically, although the Volvo was felt to be slightly superior on some dimensions. They also found that the readers of Consumer Reports who owned a Volvo reported having somewhat fewer mechanical problems than owners of Saabs.

They were about to go and strike a bargain with the Volvo dealer when Mr. Caldwell remembered that they had two friends who owned a Saab and one who owned a Volvo. Mr. Caldwell called up the friends. Both Saab owners reported having had a few mechanical problems but nothing major. The Volvo owner exploded when asked how he liked his car. “First that fancy fuel injection computer thing went out: $500 bucks. Next I started having trouble with the rear end and had to replace it and then the transmission and the clutch. I finally sold it after 3 years for junk.”

Given that the Caldwells are going to buy either a Volvo or a Saab today, in order to save $2500, which do you think they should buy?

1. They should definitely buy the Saab
2. They should probably buy the Saab
3. **They should probably buy the Volvo**
4. **They should definitely buy the Volvo**
5. *Sample Size: Hospital Problem*

A certain town is served by two hospitals. In the larger hospital about 45 babies are born each day, and in the smaller hospital about 15 babies are born each day. As you know, about 50 percent of all babies are boys. However, the exact percentage varies from day to day. Sometimes it may be higher than 50 percent, sometimes lower. For a period of 1 year, each hospital recorded the days on which more than 60 percent of the babies born were boys.

Which hospital do you think recorded more such days?

a) The larger hospital

b) **The smaller hospital**

c) About the same (that is, within 5 percent of each other)

*3) Sample Size: Squash Problem*

‘As you know, a game of squash can be played either to 9 or to 15 points. Holding all other rules of the game constant, if A is a better player than B, witch scoring system will give A a better chance of winning?’

a) 9 points

b) **15 points**

*4) Regression to the mean*

After the first 2 weeks of the major league baseball season, newspapers begin to print the top 10 batting averages. Typically, after 2 weeks, the leading batter often has an average of about .450. However, no batter in major league history has ever averaged .450 at the end of the season.

Why do you think this is? Choose one:

a) When a batter is known to be hitting for a high average, pitchers bear down more when they pitch to him.

b) Pitchers tend to get better over the course of a season, as they get more in shape. As pitchers improve, they are more likely to strike out batters, so batters’ averages go down.

**c) A player’s high average at the beginning of the season may be just luck. The longer season provides a more realistic test of a batter’s skill.**

d) A batter who has such a hot streak at the beginning of the season is under a lot of stress to maintain his performance record. Such stress adversely affects his playing.

e) When a batter is known to be hitting for a high average, he stops getting good pitches to hit. Instead, pitchers “play the corners” of the plate because they don’t mind walking him.

*5) Gambler’s Fallacy 1*

The slot machine problem:

“When playing slot machines, people win something about 1 in every 10 times. Julie, however, has just won on her first three plays.

What are her chances of winning the next time she plays?

____ out of 10

Answer:

The correct response, 1 out of 10, was scored as correct, and all other responses were scored as incorrect.

*6) Gambler’s Fallacy 2*

The coin problem:

Imagine that we are tossing a fair coin (a coin that has a 50/50 chance of coming up heads or tails) and it has just come up heads 5 times in a row. For the 6th toss do you think that:

a) It is more likely that tails will come up than heads.

b) It is more likely that heads will come up than tails.

c) **Heads and tails are equally probable on the sixth toss.**

*7) Conjunction problem*

Linda is 31 years old, single, outspoken, and very bright. She majored in philosophy. As a student, she was deeply concerned with issues of discrimination and social justice, and also participated in "Occupy Wall Street" demonstrations.

Please rank the following possibilities in terms of likelihood. (That is, rank them by pulling the most likely options to the top and pulling the least likely to the bottom)

Linda is a teacher in elementary school.

Linda works in a bookstore and takes Yoga classes.

Linda is active in the feminist movement.

Linda is a psychiatric social worker.

Linda is a member of the League of Women Voters.

Linda is a bank teller.

Linda is an insurance salesperson.

Linda is a bank teller and is active in the feminist movement.

Answer: Responses indicating that the conjunction was more likely than one of its components were incorrect and scored as 0, and all other responses were scored as 1.

*8) Covariation Detection*

A doctor had been working on a cure for a mysterious disease. Finally, he created a drug that he thinks will cure people of the disease. Before he can begin to use it regularly, he has to test the drug. He selected 300 people who had the disease and gave them the drug to see what happened. He selected 100 people who had the disease and did not give them the drug in order to see what happened. The table below indicates what the outcome of the experiment was:

|  | **Cure** | |
| --- | --- | --- |
|  | **Yes** | **No** |
| **Treatment present** | 200 | 100 |
| **Treatment absent** | 75 | 25 |

Was the treatment positively or negatively associated with the cure for this disease? Indicate your answer by choosing the appropriate number on the following scale:

−10 (strong negative association) to +10 (strong positive association)

Answer:

Negative judgments, which indicated the inefficacy of the treatment, were scored as correct.

*9) Methodological Reasoning*

The city of Middleopolis has had an unpopular police chief for a year and a half. He is a political appointee who is a crony of the mayor, and he had little previous experience in police administration when he was appointed.

The mayor has recently defended the chief in public, announcing that in the time since he took office, crime rates decreased by 12%.

Which of the following pieces of evidence would most deflate the mayor's claim that his chief is competent?

(a) **The crime rates of the two cities closest to Middleopolis in location and size have decreased by 18% in the same period.**

(b) An independent survey of the citizens of Middleopolis shows that 40% more crime is reported by respondents in the survey than is reported in police records.

(c) Common sense indicates that there is little a police chief can do to lower crime rates. These are for the most part due to social and economic conditions beyond the control of officials.

(d) The police chief has been discovered to have business contacts with people who are known to be involved in organized crime.

*10) Bayesian Reasoning*

*Step 1:* You were told that David Maxwell attended a party in which 25 male university professors and 75 male business executives took part, 100 people all together.

*Question:* What do you think the probability is that David Maxwell is a university professor? (0-100)

*Step 2:* You were told that David Maxwell is a member of the Bear's Club. 70% of the male university professors at the above mentioned party were members of the Bear's Club, and 90% of the male business executives at the party were members of the Bear's Club.

*Question:* What do you think the probability is that David Maxwell is a university professor? (0-100)

Answer: Any decrease in probability estimate for Step 2 relative to Step 1 was scored 1. All increases were scored 0 (incorrect).

*11) Probabilistic reasoning: Denominator neglect*

Assume that you are presented with two trays of black and white marbles: a large tray that contains 100 marbles and a small tray that contains 10 marbles. The marbles are spread in a single layer on each tray.

You must draw out one marble (without peeking, of course) from either tray. If you draw a black marble, you win $2.

Consider a condition in which the small tray contains 1 black marble and 9 white marbles, and the large tray contains 8 black marbles and 92 white marbles. From which tray would you prefer to select a marble in a real situation?

1. **Small tray (10 marbles)**
2. Large tray (100 marbles)

*12) Probability Matching*

A die with 4 red faces and 2 green faces will be rolled 60 times. Before each roll you will be asked to predict which color (red or green) will show up once the die is rolled. You will be given one dollar for each correct prediction. Assume that you want to make as much money as possible.

What strategy would you use in order to make *as much money as possible* by making the most correct predictions?

Strategy A: Go by intuition, switching when there has been too many of one color or the other.

Strategy B: Predict the more likely color (red) on most of the rolls but occasionally, after a long run of reds, predict a green.

Strategy C: Make predictions according to the frequency of occurrence (4 of 6 for red and 2 of 6 for green). That is, predict twice as many reds as greens.

**Strategy D: Predict red on all of the 60 rolls.**

Strategy E: Predict green on all of the 60 rolls.

Strategy F: Predict more red than green, but switching back and forth depending upon “runs” of one color or the other.

*13) Sunk Cost*

(1)You are staying in a hotel on vacation. You paid $6.95 to see a movie on pay T.V.

After 5 minutes, you are pretty bored with the film.

Would you watch the movie or not?

(2) You are staying in a hotel on vacation. You turn on the T.V. and there is a movie on. After 5 minutes, you are pretty bored with the film.

Would you watch the movie or not?

Answer:

Responses were scored as correct if the participant consistently chose across the two situations (either continuing to watch the movie in both cases, or switching to another channel in both cases), and as incorrect if the participant displayed a sunk cost (that is, continuing to watch the movie if it had been paid for but not if it was free).

*14) Outcome Bias 1*

A 55-year-old man had a heart condition. He had to stop working because of chest pain. He enjoyed his work and did not want to stop. His pain also interfered with other things, such as travel and recreation. A type of bypass operation would relieve his pain and increase his life expectancy from age '65 to age 70. However, 8% of the people who have this operation die from the operation itself.

Case 1: His physician decided to go ahead with the operation. The operation succeeded. Evaluate the physician's decision to go ahead with the operation.

Evaluate this decision on the following 7-point scale:

3 = clearly correct, and the opposite decision would be inexcusable;

2 = correct, all things considered;

1 = correct, but the opposite would be reasonable too;

0 = the decision and its opposite are equally good;

-1 = incorrect, but not unreasonable;

-2 = incorrect, all things considered;

-3 = incorrect and inexcusable.

Case 2: His physician decided to go ahead with the operation. The operation failed and the man died. Evaluate the physician’s decision to go ahead with the operation.

Evaluate this decision on the following 7-point scale:

3 = clearly correct, and the opposite decision would be inexcusable;

2 = correct, all things considered;

1 = correct, but the opposite would be reasonable too;

0 = the decision and its opposite are equally good;

-1 = incorrect, but not unreasonable;

-2 = incorrect, all things considered;

-3 = incorrect and inexcusable.

In case 1, participants were told about a 55 year-old man who had a heart condition and whose operation succeeded. The probability of mortality from surgery was 8%. Participants responded on a 7-point scale ranging from 1 (incorrect, a very bad decision) to 7 (clearly correct, an excellent decision).

In case 2, participants evaluated a different decision to perform surgery on a patient with a hip condition that was designed to be objectively better than the first (2% chance of death rather than 8%), even though it had an unfortunate negative outcome (death of the patient).

If participants rate the decision on the positive outcome case as better than the negative outcome decision, then they have displayed outcome bias. The absence of outcome bias was scored as the correct response for this problem.

**Cognitive ability measures**

**Wordsum**

Source: Huang & Hauser (1998)

We would like to know something about how people go about guessing words they do not know. On this card are listed some words. You may know some of them, and you may not know quite a few of them. On each line, the first word is in capital letters--- like BEAST. Then there are five other words. Please choose the word that comes closest to the meaning of the word in capital letters. For example, if the word in capital letters is BEAST, you would choose “animal” because it comes closer to BEAST than any of the other words.

Choose only one number for each item below.

EXAMPLE

BEAST 1. afraid 2. words 3. large 4. animal 5. separate 6. don’t know

A. SPACE 1. school 2. noon 3. captain 4. room 5. board 6. don’t know

B. BROADEN 1. efface 2. make level 3. elapse 4. embroider 5. widen 6. don’t know

C. EMANATE 1. populate 2. free 3. prominent 4. rival 5. come 6. don’t know

D. EDIBLE 1. auspicious 2. eligible 3. fit to eat 4. sagacious 5. able to speak 6. don’t know

E. ANIMOSITY 1. hatred 2. animation 3. disobedience 4. diversity 5. friendship 6. don’t know

F. PACT 1. puissance 2. remonstrance 3. agreement 4. skillet 5. pressure 6. don’t know

G. CLOISTERED 1. miniature 2. bunched 3. arched 4. malady 5. secluded 6. don’t know

H. CAPRICE 1. value 2. a star 3. grimace 4. whim 5. inducement 6. don’t know

I. ACCUSTOM 1. disappoint 2. customary 3. encounter 4. get used to 5. business 6. don’t know

J. ALLUSION 1. reference 2. dream 3. eulogy 4. illusion 5. aria 6. don’t know

H. AUDACIOUS 1. Daring 2. Smart 3. Brave 4. Loud 5. Outgoing 6. Don’t know

I. ENCUMBER  1. Impede 2. Oppress 3. Gather 4. Press 5. Encompass 6. Don’t know

**Numeracy Scale**

Source: Schwartz, Woloshin, Black, & Welch (1997)

1. Imagine that we flip a fair coin 1,000 times. What is your best guess about how many times the coin would come up heads in 1,000 flips? ____times out of 1,000.
2. In the BIG BUCKS LOTTERY, the chance of winning a $10 prize is 1%. What is your best guess about how many people would win a $10 prize if 1000 people each buy a single ticket to BIG BUCKS?____person(s) out of 1,000.
3. In ACME PUBLISHING SWEEPSAKES, the chance of winning a car is 1 in 1,000. What percent of tickets to ACME PUBLISHING SWEEPSAKES win a car?____%.

**References**

De Neys, W., & Glumicic, T. (2008). Conflict monitoring in dual process theories of thinking. *Cognition, 106*, 1284-1299.

Frederick, S. (2005). Cognitive reflection and decision making. *The Journal of Economic Perspectives, 19*, 25-42.

Huang, M. H. & Hauser, R. M. (1998). Trends in Black-White test score differentials: II The WORDSUM vocabulary test. In U. Neisser (Ed.). *The rising curve: Long-term gains in IQ and related measures* (pp.303-332). Washington DC: American Psychological Association.

Schwartz, L. M., Woloshin, S., Black, W. C., & Welch, H. G. (1997). The role of numeracy in understanding the benefit of screening mammography. *Annals of Internal Medicine, 127*, 966-972.

Toplak, M. V., West, R. F., & Stanovich, K. E. (2011). The Cognitive Reflection Test as a predictor of performance on heuristics-and-biases tasks. *Memory & Cognition, 39,* 1275-1289.

Toplak, M. V., West, R. F., & Stanovich, K. E. (2014). Assessing miserly information processing: An expansion of the Cognitive Reflection Test. *Thinking & Reasoning, 20*, 147-168.
