# Supplementary material for: Atheists and Agnostics Are More Reflective than Religious Believers: Four Empirical Studies and a Meta-Analysis
Source: PLoS One. 2016 Apr 7;11(4):e0153039. doi: 10.1371/journal.pone.0153039 (PMC4824409; doi:10.1371/journal.pone.0153039)
Supplement: S4 Text — (DOCX) [file pone.0153039.s004.docx]

Some participants completed the mass testing survey (religious belief, theism) and/or the pre-screen survey (religious affiliation) on the same day as the primary study session (cognitive variables). What follows is the results for those participants. These data are from the combined data set as very few participants completed the surveys on the same day. In total, 8.5% of the participants completed the mass testing survey on the same day as the primary study and 3.9% of completed the pre-screen survey on the same day as the primary study.

**Table S4.1.** Number of participants in each study who completed the mass testing or pre-screen survey on the same day as the primary study session.

|  | Study 1 | Study 2 | Study 3^a^ | Study 4 | Combined |
| --- | --- | --- | --- | --- | --- |
| Mass testing same day | 17 | 15 | 34 | 25 | 91 |
| Mass testing diff. day | 355 | 134 | 243 | 242 | 974 |
| Pre-screen same day | 9 | 8 | 18 | 7 | 42 |
| Pre-screen diff. day | 363 | 141 | 259 | 260 | 1023 |

^a^ Data on the date of completion for mass testing and pre-screen surveys was missing for 2 participants.

**Table S4.2.** Correlations (*r*) between religious belief (taken in a mass-testing survey) and performance on cognitive tests for participants who completed the cognitive variables on the same day or different day than the mass testing survey. CRT = Cognitive Reflection Test; CRT^1^ = Accuracy on original 3-item CRT; CRT^2^ = Excludes participants who indicated seeing the CRT before; CRT^3^ = Accuracy on additional CRT problems; ACS = Analytic Cognitive Style (mean of CRT^1^, CRT^3^, Base-Rate Neglect, Heuristics/Biases). *N* for each correlation listed in brackets.

|  | CRT^1^ | CRT^2^ | CRT^3^ | Base-Rate Neglect | Heuristics/Biases | ACS | Numeracy | Wordsum |
| --- | --- | --- | --- | --- | --- | --- | --- | --- |
| Combined (same day) | -.25^*^_(91)_ | -.22^*^_(89)_ | -.41^***^_(76)_ | -.40^*^_(32)_ | -.28^*^_(59)_ | -.40^***^_(91)_ | -.14 _(91)_ | -.19 _(91)_ |
| Combined (diff. day) | -.22^***^_(973)_ | -.22^***^_(938)_ | -.16^***^_(840)_ | -.23^***^_(489)_ | -.18^***^_(485)_ | -.25^***^_(974)_ | -.10^**^_(974)_ | -.17^***^_(974)_ |

***indicates *p* < .001, **indicates p < .01, *indicates p < .05.

**Table S4.3**. Mean scores on analytic cognitive style (ACS) as a function of theism, broken down by whether the cognitive variables were completed on the same day or different day than the mass testing survey. *N* for each mean is listed in brackets.

|  | | Theist | Agnostic | Atheist | ANOVA |
| --- | --- | --- | --- | --- | --- |
|  | Combined (same day) | .38_(44)_ | .43_(26)_ | .57_(21)_ | *F* = 5.15^**^ |
|  | Combined (diff. day) | .37_(526)_ | .44_(268)_ | .50_(179)_ | *F* = 18.29^***^ |
|  | ***indicates *p* < .001, **indicates p < .01, *indicates p < .05. | | | | |

**Table S4.4**. Mean scores on analytic cognitive style (ACS) as a function of religious affiliation, broken down by whether the cognitive variables were completed on the same day or different day than the pre-screen survey. *N* for each mean is listed in brackets.

|  | | Theist | None | Agnostic | Atheist | ANOVA |
| --- | --- | --- | --- | --- | --- | --- |
|  | Combined (same day) | .41_(24)_ | .44_(4)_ | 57._(6)_ | .56_(8)_ | *F* = 1.07 |
|  | Combined (diff. day) | .36_(573)_ | .41_(167)_ | .49_(136)_ | .55_(125)_ | *F* = 23.91^***^ |
|  |  | ***indicates *p* < .001, **indicates p < .01, *indicates p < .05. | | | | |
